# Cytoplasmic DDX3 as prognosticator in male breast cancer

**DOI:** 10.1007/s00428-021-03107-4

**Published:** 2021-05-11

**Authors:** Carmen C. van der Pol, Cathy B. Moelans, Quirine F. Manson, Marilot C. T. Batenburg, Elsken van der Wall, Inne Borel Rinkes, Lenny Verkooijen, Venu Raman, Paul J. van Diest

**Affiliations:** 1 Department of Surgical Oncology, Alrijne Hospital Leiderdorp, Leiderdorp, The Netherlands; 2 Departments of Pathology, University Medical Center Utrecht Cancer Center, PO Box 85500, 3508 GA Utrecht, The Netherlands; 3 Department of Radiotherapy, University Medical Center Utrecht, Utrecht, The Netherlands; 4 Department of Medical Oncology, University Medical Center Utrecht, Utrecht, The Netherlands; 5 Department of Surgical Oncology, University Medical Center Utrecht, Utrecht, The Netherlands; 6 Department of Radiology and Oncology, Johns Hopkins School of Medicine, Baltimore, MD USA

**Keywords:** Male breast cancer, Biomarker, DDX3, Prognosis

## Abstract

Male breast cancer (MBC) is a rare disease. Due to its rarity, treatment is still directed by data mainly extrapolated from female breast cancer (FBC) treatment, despite the fact that it has recently become clear that MBC has its own molecular characteristics. DDX3 is a RNA helicase with tumor suppressor and oncogenic potential that was described as a prognosticator in FBC and can be targeted by small molecule inhibitors of DDX3. The aim of this study was to evaluate if DDX3 is a useful prognosticator for MBC patients. Nuclear as well as cytoplasmic DDX3 expression was studied by immunohistochemistry in a Dutch retrospective cohort of 106 MBC patients. Differences in 10-year survival by DDX3 expression were analyzed using log-rank test. The association between clinicopathologic variables, DDX3 expression, and survival was tested in uni- and multivariate Cox-regression analysis. High cytoplasmic DDX3 was associated with high androgen receptor (AR) expression while low nuclear DDX3 was associated with negative lymph node status. Nuclear and cytoplasmic DDX3 were not associated with each other. In a univariate analysis, high cytoplasmic DDX3 (*p* = 0.045) was significantly associated with better 10-year overall survival. In multivariate analyses, cytoplasmic DDX3 had independent prognostic value (*p* = 0.017). In conclusion, cytoplasmic DDX3 expression seems to be a useful prognosticator in MBC, as high cytoplasmic DDX3 indicated better 10-year survival.

## Introduction

Male breast cancer (MBC) is a rare disease. Less than 1% of all male cancer patients have breast cancer and males account for less than 1% of all breast cancers [1]. Due to its rarity, the treatment and prediction of MBC is still directed by data that are mainly extrapolated from randomized prospective studies or clinical experience of female breast cancer (FBC) treatment, despite the fact that during the last decade more and more has become known about the unique tumor biology of MBC [1–5].Nowadays, the focus in oncology is to prevent overtreatment and gain quality of life while maintaining survival rates. The average age of male patients diagnosed with breast cancer is around 65 years, compared to 55 for female patients [4, 6–8]. Overtreatment with adjuvant chemotherapy should be avoided because of the side effects, especially at older age. Moreover, the side effects of hormonal therapy in males are significant. If enhanced survival can be predicted, better decisions can be made for personalized chemo- and hormonal therapy.

DDX3, a member of the RNA helicase family, has multiple functions in a variety of cellular biogenesis processes [9], including cell-cycle regulation, [10], translation regulation [11, 12], DNA repair, cell survival and apoptosis [13]. DDX3 can shuffle between nucleus and cytoplasm. Cytoplasmic DDX3 and nuclear DDX3 can be measured separately. The various, often opposing roles of DDX3, both nuclear and cytoplasmic [10, 14] have been studied in several cancer types. DDX3 was reported as an oncogene in Ewing sarcoma, breast cancer [15, 16] prostate cancer [14], gallbladder carcinoma as well as pancreatic ductal adenocarcinoma [17], while being tumor suppressive in melanoma [18]. Meanwhile, for lung cancer [12], colorectal cancer [19, 20], hepatocellular carcinoma [21] and oral squamous cell carcinoma [22], literature is not uniform with regard to the role of DDX3; both oncogenic as well as tumor suppressive roles have been described [20]. If DDX3 overexpression would be relevant in MBC, this could create therapeutic options because several publications have shown that DDX3 can be targeted by the small molecule RK-33 [23–25]. Nuclear DDX3 was previously described as a negative prognostic factor in FBC [25, 26], while cytoplasmic over-expression of DDX3 was found in FBC brain metastases of especially triple negative and high grade cases [16], but the prognostic value of DDX3 in MBC had not yet been studied. The aim of this study was therefore to evaluate if DDX3 might be a useful prognosticator for MBC-patients.

## Material and methods

### Patient samples

All consecutive cases of surgical breast specimens of invasive MBC from 1986–2011 were collected from five different pathology laboratories in The Netherlands: St Antonius Hospital Nieuwegein (n = 41), Diakonessenhuis Utrecht (n = 34), University Medical Center Utrecht (n = 29), Gelre hospital Apeldoorn (n = 17) and Laboratory for Pathology East Netherlands (n = 40). Pathology reports were used to extract age, tumor size, and lymph node status. Cases with isolated tumor cells in the sentinel node were coded as lymph node negative. Grade, according to the modified Bloom and Richardson score [27], Mitotic Activity Index (MAI), histologic subtype, Ki67 (low <  = 10), androgen (AR), estrogen (ER; positive >  = 1%) [28] and progesterone receptor (PR; positive >  = 10%) and human epidermal growth factor receptor 2 (HER2) status were obtained from previous studies [2, 3, 29, 30]. The MBC tissue blocks were gathered and tissue arrays were constructed as described before [2]. In short, hematoxylin and eosin stained slides were used to identify representative tumor areas. From these areas three 0.6-mm punch biopsies from formalin-fixed and paraffin-embedded tissue blocks were obtained and embedded in a recipient paraffin block, using a precision tissue array instrument (Beecher Instruments).

Follow-up data were obtained anonymously through the Comprehensive Cancer Center of The Netherlands (IKNL) as well as by retrieving data from the Dutch central pathology administration system PALGA. The most recent date of information was used as date of last follow-up. Recurrence was coded positive if described in the follow-up data and/or proven by pathologic investigation. Overall survival (OS) was defined as the interval from surgery to death from any cause or date of lost to follow-up. For the latter, the date of the latest pathology report was used, which could concern recurrence or distant metastasis or death or benign pathologic findings. It was possible to analyze DDX3 in 106 patients of whom follow-up data were available. For this study only anonymous archival leftover pathology material was used. Therefore no informed consent was required according to Dutch legislation, which uses an opt-out system.

### Immunohistochemistry

As before [16], four µm thick sections were cut, mounted on Surgipath X-tra adhesive slides (Leica Biosystems, Milton Keynes, UK), deparaffinized in xylene and rehydrated in decreasing ethanol dilutions. Endogenous peroxidase activity was blocked with 1.5% hydrogen peroxide buffer for 15 min, followed by antigen retrieval by boiling for 20 min in EDTA buffer (pH 9.0). Slides were blocked with protein block from Novolink Polymer Detection System (Leica Microsystems, Eindhoven, The Netherlands) and subsequently incubated in a humidified chamber for 1 h with anti-DDX3 (1:50, mAb AO196) [31]. Post primary block, secondary antibodies and diaminobenzidine treatment were performed with the same Novolink Polymer Detection System according to the manufacturer’s instructions. The slides were lightly counterstained with hematoxylin and mounted. Appropriate positive and negative controls were used throughout. Nuclear and cytoplasmic DDX3 staining was scored by consensus of two observers (PvD and CvdP) and interpreted according to methods described earlier [16, 23, 32–34]. In short, the percentage of DDX3-positive nuclei was scored. Samples with ≥ 1% DDX3 staining were regarded positive. Cytoplasmic DDX3 was scored semiquantitatively as absent (0), low (1), moderate (2) or strong (3). Cases with score 0–2 were classified as having low DDX3 expression and evaluated against cases with strong expression. For comparison, DDX3 expression data of ER + /HER2- female breast cancer patients were taken from our earlier research [25, 35].

### Statistics

Statistical analyses were performed with SPSS IBM Statistics version 25. To assess the association between clinicopathological variables and DDX3 expression in MBC patients, and to compare MBC with female breast cancer patients, Pearson Chi-square- and Fisher’s exact tests were performed. For survival analysis, Kaplan–Meier curves were plotted with stratification for DDX3 expression (high/low). Differences in 10-year survival were compared with the logrank test. Cox uni- and multivariate regression analysis (enter-method) was used to assess the clinicopathological factors as well as DDX3 associated with 10-year survival. The factors that reached statistical significance in univariate analysis were used in multivariate analysis. Significance was defined as p < 0.05.

## Results

As shown in Table 1, the median age of the 106 patients 66.5 years. Most men were treated for T1-2 tumors (76.4%) and there were 23 patients with T4 breast cancer (21.7%). The vast majority (93.4%) of tumors were of no special type (formerly “ductal”; 96 ductal, 1 neuro endocrine and 2 ductulolobular) and there were one adenoid cystic, one invasive lobular carcinoma and 5 (4,7%) other invasive types (2 mucinous, 1 inverted papillary, 1 intracystic papillary and 1 cribriform cancer). 92.5% of the patients were ER positive, 64.2% PR positive and 95.3% HER2 negative. AR was noted for 71 patients of whom 34 were positive (48%). Defining breast cancer intrinsic subtypes by quantitative receptor expression showed 73.6% (n = 78) luminal A (ER + or PR + , HER2-, MIB1 < 14%), 17.9% (n = 19) luminal B (ER + or PR + , HER2 ± , MIB1 ≥ 14%), 0% HER2 driven and 2.8% (n = 3) basal-like (ER-/PR-/HER2-) cancers while 6 cases could not be defined because of missing data. The AR positive patients were mainly ER + /PR + /HER2- (70.2%) or ER + /PR-/HER2- (26.3%), while only 3.5% ER + /PR-/HER2 + and none ER + /PR + /HER2 + or triple negative.

**Table 1:**
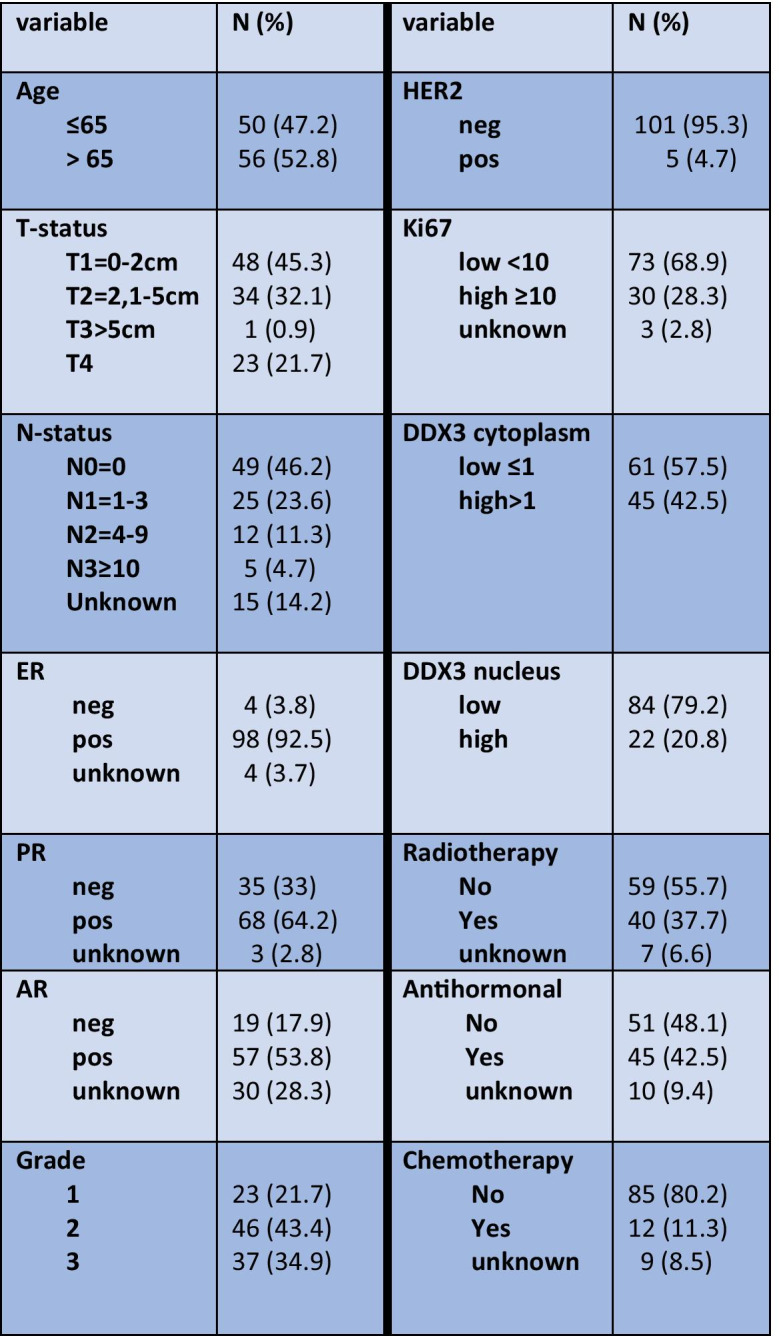
Baseline characteristics of male breast cancer patients studied for expression of the RNA helicase DDX3

Lymph node status was mostly N0 (46.2%) or N1 (23.6%). Most patients underwent a radical mastectomy (n = 91), while 5 patients were treated with breast conserving treatment. Twelve patients received adjuvant chemo- and 45 adjuvant antihormonal treatment. No neoadjuvant chemotherapy was administered.

### Associations between DDX3 expression and clinicopathologic variables

**Fig. 1:**
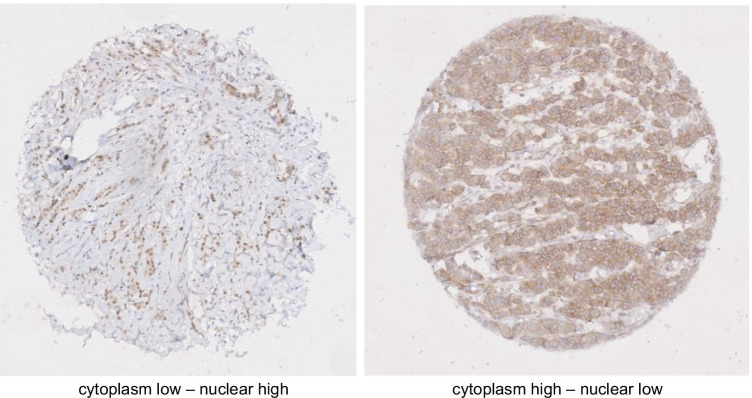
Representative examples of nuclear (left) and cytoplasmic (right) staining of DDX3 in male breast cancer

DDX3 expression was seen in the cytoplasm and the nucleus. Figure 1 shows representative examples. In 61 patients cytoplasmic DDX3-expression was low (57.5%) while the majority of cases displayed low nuclear DDX3 (79.2%). As shown in Table 2, low nuclear DDX3 was significantly associated with negative lymph node status and AR positivity was significantly associated with high cytoplasmic DDX3. ER, PR and HER2 status showed no significant associations with nuclear or cytoplasmic DDX3 expression. Nuclear and cytoplasmic DDX3 expression were not mutually exclusive and not significantly associated (Table 3).

**Table 2:**
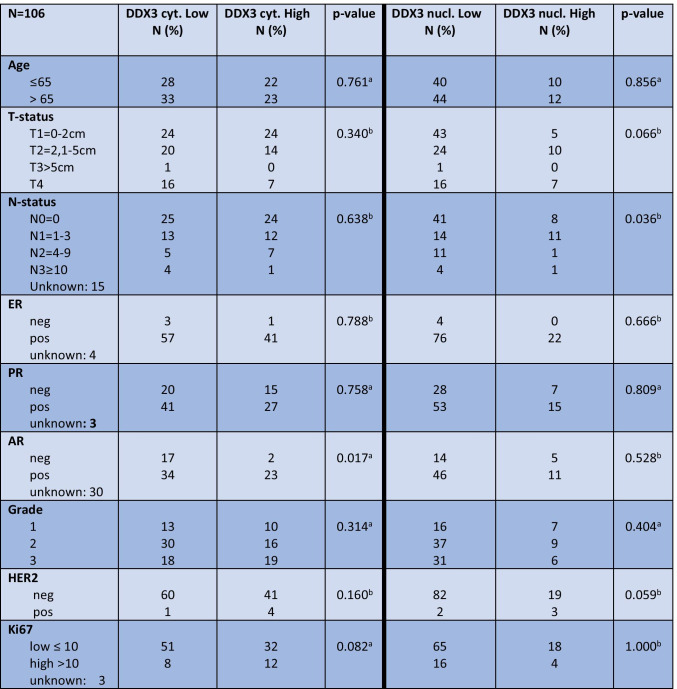
Correlations between cytoplasmic and nuclear expression of the RNA helicase DDX3 and clinicopathologic variables in male breast cancer

*a*, Chi-Square test; *b*, Fisher’s exact test

**Table 3:**
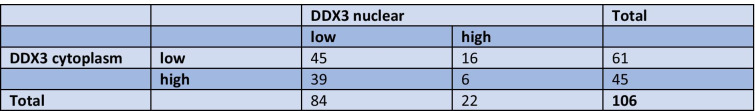
Correlation between nuclear and cytoplasmic expression of the RNA helicase DDX3 in male breast cancer

### Comparison between DDX3 expression in male and female breast cancer

MBC-patients had higher cytoplasmic (p = 0.005) and lower nuclear DDX3 (p = 0.003) levels compared to female breast cancer patients.

### Survival analysis

**Fig. 2:**
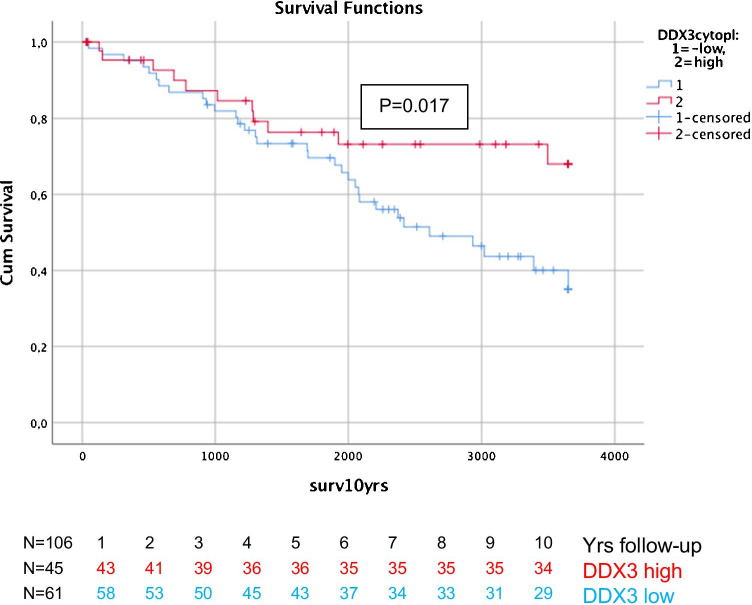
Ten-year survival curves of male breast cancer patients with low and high cytoplasmic DDX3 expression, showing better survival for patients with high cytoplasmic DDX3

Mean overall survival (OS) was 7 years, with a median of 5.7 years (range 29 days – 25 years). 10-year OS was 60.4% with a median of 5.7 year. In univariate survival analysis, cytoplasmic DDX3 (p = 0.045), age, T- and PR-status were statistically significant prognosticators (Table 4), older age, higher T-stage, negative PR-status and low cytoplasmic DDX3 expression being unfavorable. Figure 2 shows the Kaplan Meier curves for patients with low and high cytoplasmic DDX3. In multivariate analyses, cytoplasmic DDX3 expression appeared to have independent prognostic value for 10 year survival (p = 0.017) (Table 4). Nuclear DDX3 expression had no prognostic value in uni- or multivariate survival analysis.

**Table 4:**
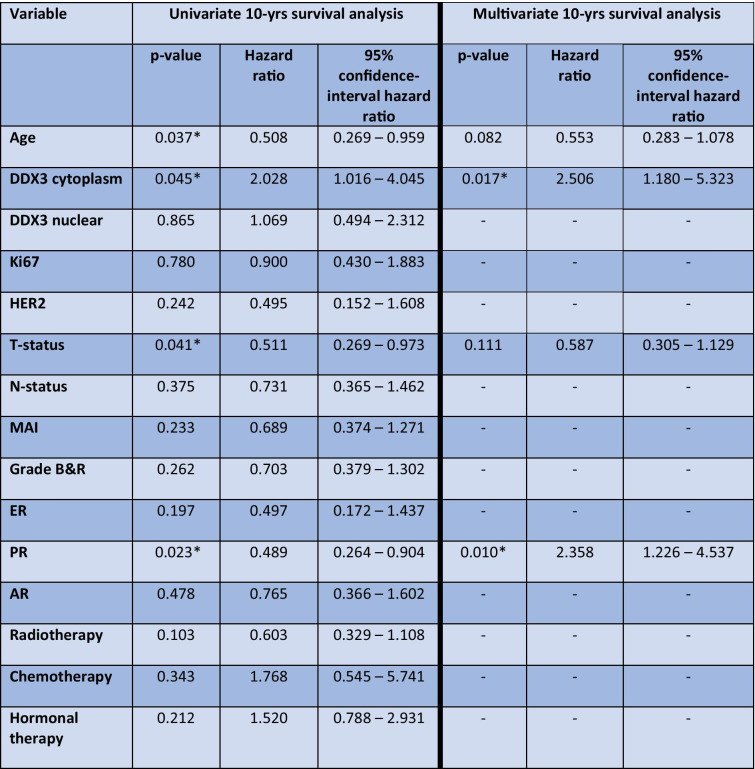
Univariate and multivariate 10-year survival analysis results of male breast cancer patients

^*^Indicates *p* < 0.05

## Discussion

This is the first report on the prognostic value of DDX3 in MBC, indicating that cytoplasmic DDX3 seems to be a useful prognostic biomarker; low expression being associated with a more unfavorable outcome.

The present results are at variance from data on DDX3 expression in FBC, where high nuclear DDX3 in the primary tumor was described as an independent predictor of worse survival [15, 16] and cytoplasmic DDX3 was not. For metastases in FBC, on the contrary, cytoplasmic DDX3 was found to be overexpressed especially for the more aggressive types like the triple negatives and grade 3 [16]. Low nuclear DDX3 in our study was associated with favorable N-status (Table 2), but of no statistically significant influence on survival. In MBC it is plausible that the oncogenic functions of DDX3 are not as evident compared to the tumor suppressive role of DDX3 [25]. Also, DDX3 has two different phenotypes, unstable and stable and the gene can have mutations in some specific sites in some types of cancer [10]. For prostate cancer, different cytoplasmic expression levels were described as well, but no association was found with survival [36].

DDX3 is a DEAD-box-RNA-helicase involved in several biogenesis cell-activities [9–13, 37]. In the literature DDX3 is described to be predominantly localized in the cytoplasm [10]. DDX3 shuttles between cytoplasm and nucleus in most human tissues and cell lines [32]. In the nucleus, DDX3 has roles in transcription, splicing, and nuclear export [10]. Cytoplasmic DDX3 moreover acts as a translation regulator and may influence cell division and/or cell growth [10, 14, 15]. Brennan studied the nucleo-cytoplasmic shuttling of DDX3 and confirmed that an N-terminal conserved Nuclear Export Signal is required for export of human DDX3 from the nucleus. Three regions were identified within DDX3 that can independently facilitate its nuclear import [10]. Different protein-binding-complexes play a role in movement of DDX3 from cytoplasm to nucleus and vice versa [10, 11, 16, 20, 35, 38]. These protein-binding-complexes appear to be different for different tumor types. Association of high nuclear DDX3 and worse prognosis has been attributed to disturbed export of DDX3 from nucleus to cytoplasm, rather than elevated import of DDX3 into the nucleus [10]. It has also been described that DDX3 subcellular localization is cell cycle dependent; more cytoplasmic in G0/1- and S-phase, and more nuclear in G2/M phase [10]. This might be a reasonable explanation for our finding that elevated cytoplasmic DDX3 is associated with a better prognosis: more cells in G0/1- or S-phase matches with most MBC being luminal A and grade 2 [1, 39]. Indeed, almost 70% was N0-N1 (Table 1) and 32/44 cases with high cytoplasmic DDX3 expression in the present study had a low Ki67 index (Table 2).

There is no convincing gender-specific explanation for the difference in prognostic value of DDX3 between MBC- and FBC-patients. Zhao, however, found that the expression level of DDX3 in hepatocellular carcinoma (HCC) was gender related and that the tendency of DDX3 down-regulation in HCC was more frequently found in males than in females [20]. It remains unclear if this concerned nuclear- and/or cytoplasmic DDX3 expression. In human there are 2 types of DDX3; DDX3X and DDX3Y, located on respectively the X- and Y-chromosomes. DDX3X and DDX3Y are 92% homologous. DDX3Y is especially important for spermatogenesis and male fertility [40], but also dual correlation of DDX3Y with cancer patient survival in different cancer types has been described [18]. Whether DDX3Y acts as a functional substitute for the loss of DDX3X in some contexts, remains unclear [41]. Lin suggests that gender differences could be explained by the fact that DDX3, located on the X-chromosome, is preferentially mutated in males [25]. If DDX3 can escape X-inactivation, females may be protected from complete functional loss by a single gene mutation. Besides this, in the same study, a possible explanation of a lower DDX3 level in males was suggested by the fact that DDX3 expression is closely associated with living habits, including smoking, alcohol consumption and other habits which are more frequent in males than in females [17]. The literature on the effect of smoking on DDX3 levels and the association with prognosis, however, is not uniform [22, 42]. Chang described a 1.5–threefold elevated DDX3 for women compared to men in normal liver tissue [21] but not clearly specified on cytoplasmic or nuclear DDX3. Trying to further explain our results, we compared cytoplasmic- and nuclear DDX3 of MBC patients of the current cohort with the ER + /HER2- FBC patients of our earlier research [25, 35], including ER-positive and HER2-negative tumors. Statistically significant gender specific differences were found (p = 0.005); MBC-patients had higher levels of cytoplasmic DDX3 and lower nuclear DDX3 compared to FBC patients.

Androgen receptor expression was statistically significant associated with cytoplasmic DDX3 in this study. High AR in ER-positive FBC patients was associated with a better prognosis in a previous study [43] and thus is in line with our findings. This is an interesting finding as in prostate cancer, especially in ﻿castration-resistant prostate cancer, high cytoplasmic DDX3 expression was associated with lower AR expression [44]. Either different DDX3 roles in different types of cancer, oncogenic or suppressive, or the small amount of AR measured MBC patients in this study, could be an explanation for these differences.

Limitations of this study are the wide range in date of diagnosis and treatment between the first and last patient included in this cohort and the relatively small cohort size. However, MBC is a rare disease and our cohort is comparable to the literature; most of the tumors are ER-positive, HER2 negative and Ki67% low and grade 2 [1, 39]. Also consistent with the literature is the first presentation in a more advanced stage; in our cohort 21.7% had a T4 at presentation [6, 7]. Our analysis was further limited by not having full insight into the compliance on adjuvant therapy whereas adherence to antihormonal treatment in men is expected to be low, knowing that for FBC patients non-compliance descending to 50% in 4 year has been described [45].

In conclusion, DDX3 is a multifunctional protein and the regulatory mechanisms and signaling pathways of DDX3 are disease specific. Although the exact mechanism of action of DDX3 in MBC is not clear, cytoplasmic DDX3 expression seems to be a useful prognosticator in MBC, high cytoplasmic DDX3 indicating better 10-year overall survival associated with low proliferation. Thereby, our results rather support a tumor suppressor role of DDX3 in MBC.
